# Socio-behavioral determinants of early vs. long-term HIV infection in Kazakhstan: roles of self-testing, alcohol, drug use and sexual networks

**DOI:** 10.3389/fpubh.2025.1697913

**Published:** 2025-10-28

**Authors:** Marat Kuzembayev, Lyudmila S. Yermukhanova, Saeed Sadeghi, Gulshara Aimbetova, Aisha Urazayeva, Gulbanu Arynova, Alireza Afshar

**Affiliations:** ^1^Department of Public Health and Public Health Care, West-Kazakhstan Marat Ospanov Medical University, Aktobe, Kazakhstan; ^2^Department of Public Health, Asfendiyarov Kazakh National Medical University, Almaty, Kazakhstan; ^3^Department of Epidemiology, West-Kazakhstan Marat Ospanov Medical University, Aktobe, Kazakhstan; ^4^Department of Epidemiology, Biostatistics and Evidence-Based Medicine, Al-Farabi Kazakh National University, Almaty, Kazakhstan

**Keywords:** acquired immunodeficiency syndrome, human immunodeficiency virus, HIV infections/diagnosis, alcohol drinking, self testing, retrospective studies

## Abstract

**Background:**

Human immunodeficiency virus (HIV) remains a major global public health challenge, with eastern Europe and central Asia experiencing a rapid rise in new infections. In Kazakhstan, structural barriers and substance use complicate prevention and care. This study aimed to determine the socio behavioral determinants of recent vs. long term HIV infection and identify predictors of baseline viral load and CD4 count among newly diagnosed adults.

**Methods:**

We conducted a retrospective cross-sectional study of 452 adults diagnosed with HIV in 2023–2024 at the Kostanay Regional Center for Prevention and Control of AIDS. Clinical data were extracted from medical records and behavioral information collected via structured questionnaires. Infection stage was defined as recent (< 6 months) or long term (>6 months). Logistic regression identified factors associated with infection stage; linear regression assessed predictors of log10 viral load and CD4 count.

**Results:**

Participants were predominantly married (63.3%) and middle aged (mean 41.5 years). Self reported alcohol (80.8%) and drug use (67.5%) were common, and 80.8% had used an HIV self test prior to diagnosis. Early infection declined from 27.4% in 2023 to 16.2% in 2024. Logistic models showed that male gender, any alcohol use and lifetime drug use were associated with higher odds of recent infection, while each additional sexual partner increased the odds of long term infection. Early cases were more likely to have self tested and to report recent sexually transmitted infections. Older age predicted higher viral load and lower CD4 count, whereas recent infection was associated with better immunological status.

**Conclusion:**

Early detection is occurring among substance users through self testing and harm reduction programs, yet older adults and those with multiple partners remain at risk for delayed diagnosis. Integrated interventions that couple self testing with substance use services and targeted outreach to older individuals could accelerate progress toward epidemic control.

## 1 Introduction

The human immunodeficiency virus (HIV) remains a formidable public-health challenge, with roughly 75–80 million people infected and 32–40 million deaths since the epidemic's emergence (1). In 2014, there were roughly 2 million new HIV infections and 36.9 million people living with the virus. By 2018, the number of people living with HIV increased to 37.9 million with 1.7 million new infections that year, and by 2020 and 2022, it further rose to 37.7 million and 39 million, respectively (1, 2). Despite global efforts that have reduced AIDS mortality by more than 40% since the mid-2000s, about 1.3 million people acquired HIV and 630,000 died from AIDS-related illnesses in 2023 (1). Prevalence varies widely across regions, and eastern Europe and central Asia now record the fastest growth in new infections and AIDS deaths (3, 4). In this region only about two-thirds of people living with HIV know their status and half receive antiretroviral therapy (ART) (3). Kazakhstan typifies these challenges: national data show a 39 % increase in HIV prevalence since 2010, with high infection rates among people who inject drugs, men who have sex with men, sex workers and prisoners (3, 5). Structural factors including stigma, punitive drug policies and under-resourced health systems hamper prevention, testing and treatment (3).

Social and behavioral determinants are central to HIV epidemiology. Poverty, low education, unstable housing and unemployment constrain access to information and health services and increase vulnerability (6, 7). In South African townships, residents in informal dwellings and those without secondary education had significantly higher odds of HIV infection, while unmarried individuals were more than twice as likely to be infected (6). Marriage may offer some protection by limiting concurrency, but gender power imbalances within marriages can still expose women to infection (8, 9). Economic insecurity often drives transactional sex or impedes condom negotiation, and stigma and discrimination discourage testing and care (3). Behavioral factors intersect with these social forces: multiple and concurrent partnerships, inconsistent condom use, alcohol consumption and drug use are well-established drivers of transmission (10, 11). Alcohol and other psychoactive substances impair judgement and hamper condom negotiation, while injection drug use (IDU) remains a major transmission route—there are an estimated 15.6 million people who inject drugs globally, about 17.8 % of whom are HIV-positive (12).

Sexually transmitted infections (STIs) further amplify HIV transmission by disrupting mucosal barriers and increasing inflammation. Genital ulcers due to syphilis or herpes elevate HIV susceptibility several-fold, and non-ulcerative infections like gonorrhea and chlamydia increase viral shedding and infectiousness (13, 14). Effective STI management can reduce HIV incidence, underscoring the importance of integrated services (13). Expanding access to early diagnosis is equally critical. Innovations such as community-based testing, HIV self-testing and recency testing broaden reach beyond traditional clinics. HIV self-testing allows individuals to collect and interpret their own specimens; programs like the Self-Testing Africa initiative have distributed more than 100 million kits and demonstrated high acceptability and cost-effectiveness (15). Evidence suggests self-testing reaches men and other groups underserved by clinic-based testing and facilitates linkage to prevention and care. Recency testing distinguishes recent from long-term infections using assays such as limiting antigen avidity, enabling targeted interventions and more accurate incidence estimation (16). Initiating treatment during recent infection reduces viraemia and preserves immune function, highlighting the value of early detection (16, 17).

Nevertheless, gaps in the HIV care cascade persist. Globally, about 87 % of people living with HIV are aware of their status and 77 % are on treatment (1), leaving millions undiagnosed or untreated. In central Asia harm-reduction services remain limited, and socio-economic stressors such as poverty and unemployment compound substance use and risky sexual behaviors (5). Research that disentangles how demographic, behavioral and socio-economic factors influence HIV acquisition and progression is therefore imperative. Few epidemiological studies from central Asia have examined the timing of infection or how substance use, sexual behavior and socio-economic circumstances jointly shape HIV outcomes; most available data are derived from sentinel surveillance or descriptive reports that lack information on recency and associated factors (3, 5).

To address this gap, we undertook a retrospective cohort study at the Kostanay Regional Center for Prevention and Control of AIDS in Kazakhstan between January 2023 and December 2024. We reviewed medical records and interviewed 452 adults newly diagnosed with HIV to determine the proportion of recent vs. long-term infections, identify socio-behavioral factors associated with recent infection and examine predictors of viral load and CD4 count. Variables assessed included age, gender, marital status, alcohol and drug use, history of STIs, condom use, number of sexual partners, uptake of self-testing and baseline immunological markers. This study aims to assess the prevalence and temporal trends of recent vs. long-term HIV infection among newly diagnosed adults in Kostanay, Kazakhstan; to identify socio-behavioral and testing practices (including self-testing, alcohol and drug use, sexual behavior and STI history) associated with the stage of infection; and to evaluate demographic and behavioral predictors of baseline viral load and CD4 count.

## 2 Materials and methods

### 2.1 Study design and setting

This retrospective cross-sectional study was conducted at the Kostanay Regional Center for the Prevention and Control of AIDS in Kazakhstan and included individuals diagnosed with HIV infection during 2023 and 2024. The study aimed to evaluate differences in clinical, behavioral, and epidemiological indicators over time, to identify factors associated with early vs. long-term HIV infection, and to determine predictors of viral load and CD4^+^ T-cell count.

### 2.2 Study population

We included all consecutive adults (≥18 years) with a newly confirmed HIV diagnosis at the Kostanay Regional Center between 1 January 2023 and 31 December 2024, as recorded in the center's electronic registry. Diagnosis followed the national confirmatory algorithm.

Exclusion criteria were:

- Age <18 years
- Refusal to participate or withdrawal of consent
- Incomplete core diagnostic or behavioral data
- Duplicate records

A total of 452 individuals met the inclusion criteria and were analyzed. Missing data for individual variables ranged from 11.5% (CD4 count) to 11.7% (viral load).

### 2.3 Data sources

Information for this study was obtained from two main sources. Archival medical records provided clinical and laboratory information, including outpatient medical forms, viral load and CD4^+^ T-cell test results, epidemiological registration cards, and entries from the national electronic HIV surveillance database. In parallel, participants completed a structured questionnaire adapted from national HIV surveillance instruments at the time of diagnosis. This instrument collected detailed socio-demographic information, behavioral risk factors such as alcohol and drug use, sexual behaviors and condom use, HIV testing history, and recent medical history, including any sexually transmitted infection (STI) diagnoses.

### 2.4 Variable definitions

The primary classification variable, infection stage, was defined as either early HIV infection, diagnosis within 6 months of the estimated date of infection based on recency testing algorithms and patient-reported history, or long-term HIV infection, with a duration exceeding 6 months. Infection stage was abstracted from the clinic registry as recorded at diagnosis. The registry field reflects the site's standard recency classification procedure, which combines a serological antibody-avidity assessment conducted by the regional reference laboratory with programmatic exclusions (evidence of prior HIV diagnosis or ART exposure at/before testing) and clinical/testing history. For clarity, we report “recent” as ≤ 6 months, consistent with the mean duration-of-recent-infection window used in programmatic recency classification at the site. We did not perform laboratory testing ourselves, and we analyzed the recency status as recorded in the medical record.

Alcohol use was recorded as a binary variable (Yes = 1, No = 0). The quantity of alcohol consumed was expressed as mean grams of pure alcohol per day (“alcohol servings”), calculated using the formula:

Volume (ml) × Alcohol % × 0.79

One “Alcohol Servings” refers to the number of alcoholic beverages typically consumed per day, expressed in grams of pure alcohol. A standard serving equals approximately 10 grams of alcohol, based on conversion formulas such as:

- Beer (330 ml, 5% ABV): ~13 g
- Wine (140 ml, 12% ABV): ~13.3 g
- Spirits (40 ml, 40% ABV): ~12.6 g

Drug use ever referred to lifetime use of illicit substances, including both injecting and non-injecting routes. Injecting drug use and needle sharing were recorded as binary (Yes/No) variables. STI diagnosis indicated laboratory-confirmed sexually transmitted infection within the past 6 months. HIV self-testing was defined as ever using an HIV self-test prior to confirmatory diagnosis. Viral load (VL) was measured by PCR and expressed as copies/mL; it was analyzed both as a continuous log_10_-transformed variable (LG10VL) and dichotomized at >100,000 copies/mL for some analyses. CD4^+^ T-cell count was measured in cells/μL and analyzed as a continuous log_10_-transformed variable (LG10CD4). Behavioral measures included condom-use frequency, recorded on a five-point ordinal scale from “Never” to “Always,” and the number of sexual partners in the past 12 months, treated as a continuous variable.

### 2.5 Missing data and sensitivity analyses

Viral load (VL) and CD4 values were extracted as the leading numeric component from their source strings and analyzed as continuous and dichotomized variables. We summarized missingness overall and by infection stage (EARLY vs. long-term). Because missingness was extremely low in the analytic dataset (see Results), our primary analyses used complete cases. To evaluate robustness, we conducted extreme-bounds sensitivity analyses for two clinically relevant thresholds, HIGH-VL ≥ 100,000 copies/ml and LOW-CD4 < 200 cells/μl, by reassigning the few missing values to the exposure category most likely to alter the odds ratio (worst-case: EARLY = 1 → exposed, EARLY = 0 → unexposed; best-case: EARLY = 1 → unexposed, EARLY = 0 → exposed). Given only one missing value for each laboratory marker, multiple imputation was not pursued because it could not materially change estimates.

### 2.6 Outcome measures

The primary outcomes were infection stage (early vs. long-term) and the two continuous biomarker measures LG10VL and LG10CD4. Secondary outcomes included behavioral variables such as condom-use frequency and the number of sexual partners in the past year, as well as the binary high viral load measure (>100,000 copies/ml).

### 2.7 Statistical analysis

All analyses were performed using IBM SPSS Statistics, Version 29.0 (IBM Corp., Armonk, NY, USA). Descriptive statistics were reported as means ± standard deviations (SD) for continuous variables and as counts with percentages for categorical variables. Bivariate comparisons between groups were conducted using independent *t*-tests for continuous variables and chi-square or Fisher's exact tests for categorical variables.

Multivariable modeling was conducted as follows:

Hierarchical binary logistic regression to identify predictors of infection stage:

- Model 1: Demographics (age, gender)
- Model 2: Model 1 + Behavioral factors (alcohol use, drug use)
- Model 3: Model 2 + Continuous risk measures (alcohol servings/day, number of sexual partners)

Multiple linear regression to assess predictors of LG10VL and LG10CD4, adjusting for demographic and behavioral variables. Ordinal logistic regression for condom-use frequency (five ordered categories), with the proportional-odds assumption tested. Model assumptions were assessed using variance inflation factors (VIF) to detect collinearity, the Hosmer–Lemeshow test for logistic regression fit, and the proportional-odds test for ordinal models. Statistical significance was defined as two-sided *p* < 0.05.

*Post-hoc* detectability: With 54 early and 143 long-term cases (α = 0.05, two-sided), we used Power Analysis; Two Independent Proportions to approximate detectable effects for key exposures. Using the observed control prevalences (alcohol = 0.82; ever-drug = 0.35), we iteratively solved for the case prevalence yielding ~80% power and converted (*p*1, *p*_2_) to odds ratios. This approach provides a transparent detectability benchmark complementary to confidence-interval-based precision.

### 2.8 Ethical considerations

The study protocol was reviewed and approved by the Ethics Committee of West Kazakhstan Marat Ospanov Medical University (protocol approval number: No. 11.04/03). All procedures were conducted in accordance with the ethical standards of the institutional and national research committees and with the 1964 Declaration of Helsinki and its later amendments. Written informed consent was obtained from all participants prior to inclusion, permitting the use of their anonymized data for research purposes. Participant confidentiality was strictly maintained; all identifying information was removed from the dataset before analysis, and data were stored securely in password-protected systems accessible only to authorized research personnel.

## 3 Results

### 3.1 The study cohort was predominantly married, middle-aged, and reported high levels of alcohol and drug use

A total of 452 individuals with confirmed HIV infection were included in the analysis. The mean age was 41.5 ± 10.2 years, and 160 (35.4%) were male. Most participants were married (286; 63.3%), while 166 (36.7%) were single. The mean baseline viral load was 6.56 × 1,055 ± 1.65 × 1,066 copies/mL, and the mean CD4++ T-cell count was 433.5 ± 1,053.7 cells/μl.

*Post-hoc* detectability (power analysis): Given the available sample, the study achieves ~80% power to detect OR ≥ ~2.5 for ever-drug use (control prevalence ≈0.35) but is under-powered for alcohol (control prevalence ≈0.82) unless the OR is very large. See Supplementary Table S1 for counts, crude/adjusted ORs, and 80%-power thresholds.

In the analytic dataset with infection stage defined (*n* = 197; 54 EARLY, 143 long-term), VL was missing in 1 case (0.51%) and CD4 was missing in 1 case (0.51%). By stage, the single missing VL occurred in long-term infection, and the single missing CD4 occurred in EARLY infection. Using complete cases, EARLY infection was less likely to coincide with HIGH-VL ≥ 100,000 copies/ml (OR = 0.50, 95% CI 0.26–0.95, *p* = 0.04) and LOW-CD4 < 200/μl (OR = 0.07, 95% CI 0.01–0.52, *p* = 0.01). Results were stable under extreme-bounds assignments for the two missing values (HIGH-VL worst-case OR = 0.51; best-case OR = 0.49; LOW-CD4 worst-case OR = 0.14; best-case OR = 0.07). Detailed 2 × 2 counts and sensitivity scenarios are in Supplementary Table S2.

Alcohol consumption within the past year was common: 365 participants (80.8%) reported any alcohol use. Regarding drinking frequency, 190 (42.0%) consumed alcohol 2–4 times per month, 71 (15.7%) drank 2–3 times per week, and 38 (8.4%) reported drinking ≥4 times per week; 44 (9.7%) drank monthly or less, and 109 (24.1%) chose not to disclose. The mean daily alcohol intake was 173.7 ± 310.2 g of pure alcohol.

Illicit drug use was reported by 305 (67.5%), of whom 141 (97.2%) reported ever injecting drugs. Among those with a history of injection, 58 (53.7%) reported ever sharing needles. Exchange of sex for material reward was rare (3 participants; 0.7%). Any STI diagnosis within the last 6 months was reported by 26 (5.8%).

Before receiving a confirmatory HIV diagnosis, 365 (80.8%) had ever used an HIV self-test. Educational attainment was distributed as follows: 251 (55.5%) with low/no formal education, 158 (35.0%) with intermediate education, and 43 (9.5%) with higher education.

Missing data for individual variables ranged from 11.5% (CD4 count) to 11.7% (viral load).

### 3.2 From 2023 to 2024, early HIV infection became less common, while alcohol and drug use declined

**Figure 1:**
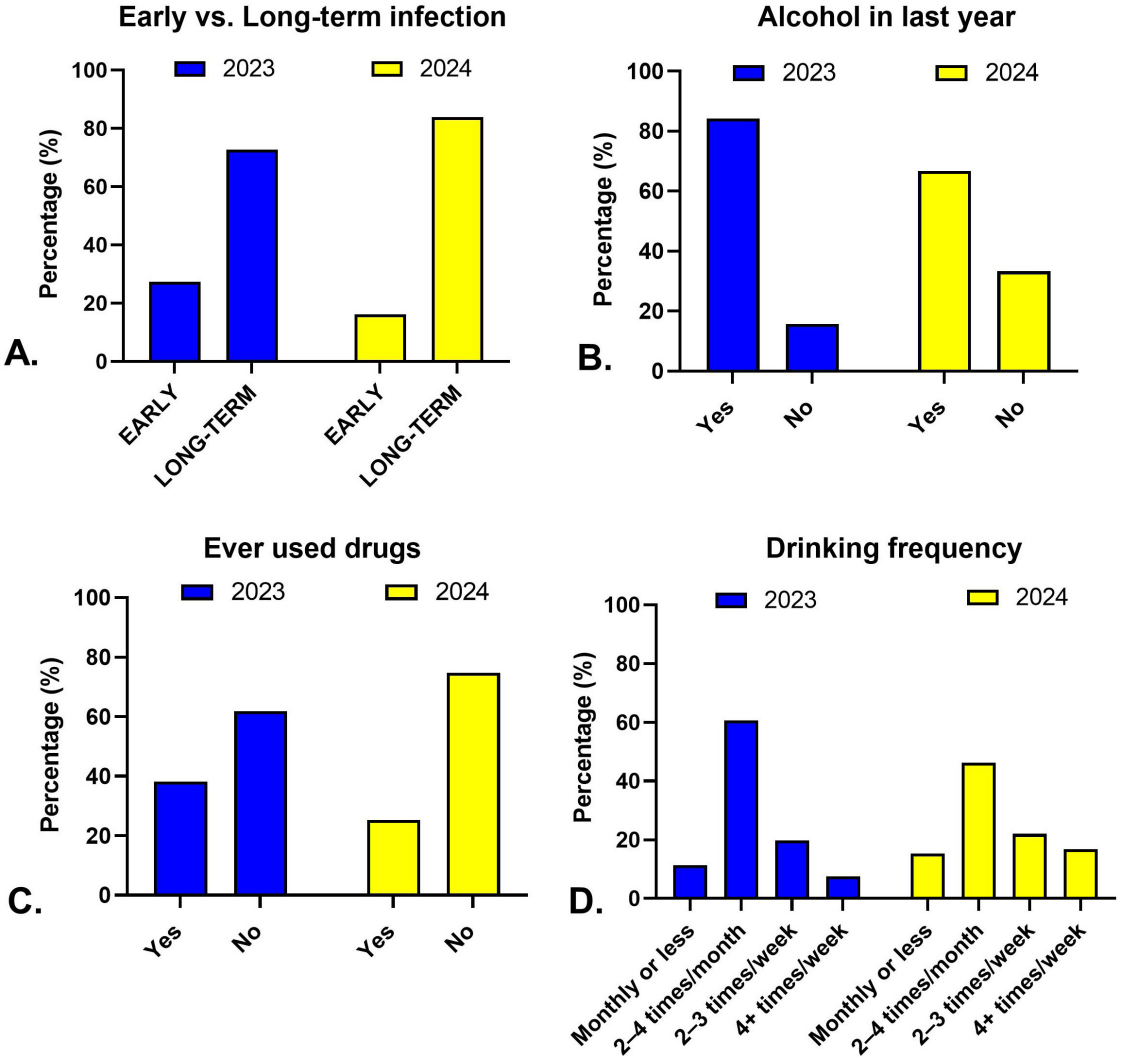
Temporal trends in infection stage and behavioral factors among HIV patients in Kazakhstan, 2023–2024. Panels illustrate changes between 2023 (blue) and 2024 (yellow) in **(A)** distribution of early vs. long-term HIV infection, **(B)** alcohol use within the past year, **(C)** lifetime drug use, and **(D)** frequency of alcohol consumption among current drinkers.

Between 2023 and 2024, the age distribution of patients shifted slightly upward, with mean age increasing from 40.8 years to 42.9 years (*p* = 0.025, Table 1). The proportion of male patients remained stable (~65%). The prevalence of early HIV infection declined from 27.4% to 16.2% (*p* = 0.007), while long-term infection increased correspondingly (Figure 1). Alcohol consumption within the past year decreased markedly from 84.3% to 66.7% (*p* < 0.001), and drinking frequency also shifted toward both lower and higher extremes, with fewer reporting 2–4 times/month and more reporting ≥4 times/week (*p* = 0.020). Ever drug use declined (38.2% to 25.3%, *p* = 0.004), but the proportion of injecting drug use within that subgroup increased (61.8% to 75.8%, *p* = 0.003). STI diagnoses within the last six months were far less common in 2024 (9.4% vs. 1.0%, *p* < 0.001). No significant changes were observed in mean viral load, proportion with VL > 100,000 copies/ml, or mean CD4 count (Table 1).

**Table 1:** Comparative analysis of clinical, behavioral, and epidemiological indicators among HIV patients in 2023 and 2024.

**Indicator/risk factor**		**2023 patients (*N* = 254)**	**2024 patients (*N* = 198)**	***p*-value (significance)**
Mean age (years)		40.81	42.89	0.025^*^
Gender (% of known cases)	Male	65.70%	63.10%	0.564
	Female	34.3%	36.9%	
Early infection (< 6 m)		27.40%	16.20%	0.007^**^
Long-term infection (>6 m)		72.60%	83.80%	
Drinking alcoholic (in last year)	Yes	84.3%	66.7%	< 0.001^***^
	No	15.7%	33.3%	
	Monthly or less	11.3%	15.2%	0.020^*^
	2–4 times a month	60.6%	46.2%	
	2–3 times a week	19.7%	22.0%	
	4 or more times a week	7.5%	16.7%	
	Don't remember or refuse to say	0.9%	0.0%	
Ever used drugs (any route)	Yes	38.2%	25.3%	0.004^**^
	No	61.8%	74.7%	
Injecting drug users (% within a subgroup)	Yes	61.8%	75.8%	0.003^**^
	No	36.6%	24.2%	
	Don't remember or refuse to say	1.6%	0.0%	
Needle sharing (% within a subgroup)	Yes	41.8%	55.6%	0.312
	No	41.8%	37.8%	
Don't remember or refuse to say	16.4%	6.7%	
Sex for material reward (ever)	Yes	1.2%	0.0%	0.125
	No	98.8%	100.0%	
Sexual partner (last 6 months)	Permanent	45.5%	54.3%	0.159
	Casual	30.6%	28.7%	
	Both	23.9%	17.1%	
Condom use at last sex with permanent partner	Yes	4.6%	3.9%	0.013^*^
	No	91.8%	85.7%	
	Don't remember or refuse to say	3.6%	10.4%	
Condom use at last sex with casual partner	Yes	8.3%	13.9%	0.336
	No	85.9%	82.5%	
	Don't remember or refuse to say	5.8%	3.6%	
Sex with known HIV + partner	Yes	27.2%	31.3%	0.591
	No	3.6%	4.2%	
	Don't remember or refuse to say	69.2%	64.6%	
Any STI diagnosis (Last 6 months)	Yes	9.4%	1.0%	< 0.001^***^
	No	90.6%	99.0%	
	Syphilis	42.1%	100.0%	0.257
	Gonorrhea	11.8%	0.0%	0.716
Reason for HIV test: Had	Symptoms	3.9%	12.0%	0.501
	Unprotected sex	2.4%	1.3%	< 0.001^***^
	Drug use	13.0%	10.0%	
	Others	80.7%	76.7%	
Mean viral load (copies/ml)		6.86 × 10^5^	6.16 × 10^5^	0.656
Viral load > 100k (copies/ml)		47.40%	62.10%	0.106
Mean CD4 count (cells/μl)		510.6	336.5	0.083

^*^p < 0.05, ^**^p < 0.01, ^***^p < 0.001.

### 3.3 Early infection was linked to more frequent self-testing and a trend toward higher condom use

Mean age and sex distribution were similar between early and long-term infection groups (Table 2). Early infection cases reported a slightly younger age at first sexual contact with men (16.96 vs. 17.88 years, *p* = 0.027). Any STI diagnosis within the last 6 months was more common among early cases (9.1% vs. 4.2%, *p* = 0.038). Symptom-driven HIV testing was less frequent in early infection (3.4% vs. 9.2%, *p* = 0.036), while self-testing before confirmatory diagnosis was reported by over half of early cases compared to less than one-third of long-term cases (*p* < 0.001). Viral load and CD4 count means did not differ significantly between groups.

**Table 2:** Comparison of clinical, behavioral, and diagnostic indicators between early and long-term HIV infection groups.

**Indicator/risk factor**		**Early infection (<6 months) (*n* = 86)**	**Long-term infection (>6 months) (*n* = 308)**	***p*-value**
Mean age (years)		41.16	42.40	0.289
Male gender (%)	Male	63.6%	64.9%	0.789
	Female	36.4%	35.1%	
Marital status	Single	58.7%	65.4%	0.174
	Married	41.3%	34.6%	
Education	No education	0.0%	0.3%	0.401
	Primary education	30.0%	70.0%	
	Secondary education	39.9%	32.7%	
	Higher education	7.7%	10.4%	
Injecting drug user (%)	Yes	28.7%	27.5%	0.304
	No	1.5%	0.3%	
	Don't remember/Don't know	69.7%	72.2%	
Shared needles (% within a subgroup)	Yes	50.0%	44.4%	0.238
	No	44.2%	37.5%	
	Don't remember or refuse to say	5.8%	18.1%	
The age of first sexual contact (mean)	With man	16.96	17.88	0.027^*^
	With woman	16.79	16.50	0.202
Sexual partner (last 6 months)	Permanent	52.0%	47.9%	0.252
	Casual	24.4%	32.4%	
	Both	23.6%	19.7%	
Condom use at last sex with casual partner	Yes	10.9%	10.9%	0.254
	No	88.0%	82.6%	
	Don't remember or refuse to say	1.1%	6.5%	
Condom use at last sex with regular partner	Yes	6.3%	3.4%	0.081
	No	91.1%	88.2%	
	Don't remember or refuse to say	2.6%	8.4%	
Any STI diagnosis (last 6 months)	Yes	9.1%	4.2%	0.038^*^
	No	90.9%	95.8%	
	Syphilis	36.4%	55.6%	0.391
	Gonorrhea	0.0%	25.0%	0.094
Sex with known HIV + partner	Yes	32.1%	27.5%	0.602
	No	3.6%	4.0%	
	Don't remember or refuse to say	64.3%	68.5%	
Reason for HIV test: Had	Symptoms	3.4%	9.2%	0.036^*^
	Unprotected sex	1.7%	2.1%	
	Drug use	15.3%	10.0%	
	Others	79.6%	78.7%	
Used HIV self-test (ever) (Before receiving a confirmed diagnosis)	Yes	51.7%	30.4%	< 0.001^***^
	No	0.7%	0.6%	
	Don't remember or refuse to say	47.6%	68.9%	
Mean viral load (copies/ml)		5.63 × 10^5^	6.98 × 10^5^	0.421
Viral load >100k (copies/ml)		1.30 × 10^5^	1.15 × 10^5^	0.643
Mean CD4 count (cells/μl)		414.5	475.6	0.571

^*^p < 0.05, ^**^p < 0.01, ^***^p < 0.001.

### 3.4 In other analyses which related to condom-use frequency, no predictor showed a strong independent association after adjustment. A borderline trend suggested that early infection cases were more likely to report frequent condom use (OR ≈ 2.9, *p* ≈ 0.06). The proportional-odds assumption was met (*p* = 0.19). Alcohol use, drug use, and gender independently predicted infection stage

In multivariable logistic regression, younger age was modestly associated with higher odds of early infection in Models 1 and 2 but not in the fully adjusted Model 3 (Table 3). In the final model, male gender (OR 0.58, 95% CI 0.36–0.93, *p* = 0.02) was associated with lower odds of long-term infection (i.e., higher odds of early infection). Alcohol use (OR 0.37, 95% CI 0.21–0.64, *p* < 0.001) and ever drug use (OR 0.48, 95% CI 0.30–0.76, *p* = 0.002) were inversely associated with long-term infection. Greater alcohol servings per day were weakly associated with higher odds of early infection (*B* = −0.001, *p* = 0.008). The number of sexual partners in the past year showed a strong positive association with long-term infection (OR 1.75 per partner, 95% CI 1.36–2.25, *p* < 0.001).

**Table 3:** Hierarchical binary logistic regression models examining the association of demographic, behavioral, and clinical predictors with the odds of long-term vs. early HIV infection.

**Predictor**	**Model 1 OR (95% CI)**	***p*-value**	**Model 2 OR (95% CI)**	***p*-value**	**Model 3 OR (95% CI)**	***p*-value**
Age (per year)	0.978 (0.958–0.997)	0.026^*^	0.978 (0.958–0.999)	0.028^*^	0.99 (0.97–1.02)	0.61
Gender (Male vs Female)	1.123 (0.739–1.707)	0.586	0.718 (0.460–1.122)	0.146	0.58 (0.36–0.93)	0.02^*^
Alcohol use (Yes vs No)	–	–	2.121 (1.257–3.579)	0.004^**^	2.73 (1.57–4.76)	< 0.001^***^
Drug use ever (Yes vs No)	–	–	1.818 (1.189–2.780)	0.002^**^	2.09 (1.31–3.33)	0.002^**^
Alcohol servings (per 10 g)	–	–	–	–	0.99 (0.98–1.00)	0.011^*^
Total partners (per partner)	–	–	–	–	1.75 (1.36–2.25)	< 0.001^***^
Constant	OR = 1.300	0.573	OR = 2.791	0.048^*^	OR = 0.417	0.201

Binary logistic regression model; outcome coded 1 = Long-term infection, 0 = Early infection. Predictors are coded 1 = Yes, 0 = No unless otherwise noted. OR < 1 indicates lower odds of Long-term infection (i.e., higher odds of Early infection), whereas OR > 1 indicates higher odds of Long-term infection. Alcohol Servings: average daily intake of pure alcohol (g/day), calculated from self-reported beverage type and quantity. All variables shown were entered simultaneously and are mutually adjusted. Two-sided p-values: ^*^p < 0.05, ^**^p < 0.01, ^***^p < 0.001.

### 3.5 Older age predicted higher viral load, while early infection predicted higher CD4 counts

In linear regression models, higher age was positively associated with log1010 viral load (*B* = 0.017, 95% CI 0.007–0.027, *p* = 0.001) after adjustment, but no other predictors reached significance (Table 4). For CD4 count, early infection was associated with higher log1010 CD4 (*B* = 0.219, 95% CI 0.124–0.313, *p* < 0.001), while older age predicted lower values (B = −0.008, 95% CI −0.012 to −0.004, *p* < 0.001). Alcohol use, drug use, alcohol servings, and gender showed no independent association with either biomarker after adjustment.

**Table 4:** Multivariable linear regression models of log10 viral load (LG10VL) and log10 CD4 count (LG10CD4) among people living with HIV. This multivariable linear regression analyses examining socio-demographic and behavioral predictors of log10 viral load and log10 CD4 count.

**Outcome variable**	**Predictor**	**B (unstandardized)**	**SE**	**95% CI (lower)**	**95% CI (upper)**	***p*-value**
LG10VL	Category (Early vs Long-term)	−0.144	0.111	−0.361	0.074	0.20
	Alcohol Use (Yes/No)	0.032	0.126	−0.216	0.280	0.80
	Drug Use Ever (Yes/No)	−0.004	0.114	−0.227	0.220	0.97
	Alcohol Servings	0.000	0.000	0.000	0.000	0.54
	Gender (Male/Female)	−0.141	0.109	−0.355	0.072	0.20
	Age (years)	0.017	0.005	0.007	0.027	0.001^**^
	Model fit	*R*^2^ = 0.037; Adj *R*^2^ = 0.024; Δ*R*^2^ = 0.028; Δ*F* (2,444) = 6.43; *p* = 0.002^**^				
LG10CD4	Category (Early vs Long-term)	0.219	0.048	0.124	0.313	< 0.001^***^
	Alcohol Use (Yes/No)	0.019	0.055	−0.088	0.127	0.73
	Drug Use Ever (Yes/No)	−0.080	0.049	−0.177	0.017	0.11
	Alcohol Servings	0.000	0.000	0.000	0.000	0.96
	Gender (Male/Female)	−0.019	0.047	−0.111	0.074	0.69
	Age (years)	−0.008	0.002	−0.012	−0.004	< 0.001^***^
	Model fit	*R*^2^=0.095; Adj *R*^2^ = 0.083; Δ*R*^2^ = 0.029; Δ*F*(2,444) = 7.15; *p* = 0.001^**^				

Predictors were selected based on prior literature and variables significant in univariate analyses.

*R*^2^ = coefficient of determination; Adj *R*^2^ = adjusted *R*^2^; Δ*R*^2^ = change in *R*^2^ after adding age and gender in Block 2. Bold model fit statistics refer to the final fully adjusted models. Age is continuous (years); gender reference = female; early infection reference = long-term infection; alcohol use and drug use coded as yes/no. ^**^ *p* < 0.01 and ^***^ *p* < 0.001.

## 4 Discussion

### 4.1 Significance of HIV cohort studies

HIV pandemic has shifted over the past four decades from an acute public health crisis to a chronic condition managed by ART. Although randomized trials have established the efficacy of ART, real-world evidence is critical to understand how demographic and behavioral factors interact with HIV acquisition, treatment uptake and disease progression. Observational cohort studies fill this gap by following diverse patient populations over time, including individuals with comorbidities or socio-economic vulnerabilities who are typically excluded from trials (18, 19). Such studies capture events not anticipated in trial protocols, generate hypotheses about social determinants of health and inform programmatic responses (18). They also enable researchers to characterize key populations and identify risk factors for HIV acquisition, which in turn informs prevention and treatment strategies (20). The present retrospective cross-sectional adds to this literature by examining newly diagnosed adults in Kazakhstan, a region experiencing rapid increases in HIV prevalence, to explore how social and behavioral determinants influence the timing of diagnosis and baseline immunological markers.

### 4.2 Sociodemographic and behavioral characteristics

Our sample was predominantly married, middle-aged and socio-economically disadvantaged, with more than half reporting low or no formal education. The mean age (41.5 years) and marriage rate (63.3 %) are consistent with Central Asian surveillance data indicating that the epidemic in this region is concentrated among adults in their 30s and 40s and increasingly affects heterosexual couples (21). Alcohol use was reported by 80.8 % of participants, with a mean daily intake of 173 g of pure alcohol-equivalent to 13–14 standard drinks. This prevalence far exceeds national estimates and underscores the centrality of alcohol consumption in the local epidemic. Meta-analysis studies of people living with HIV found that alcohol drinkers were 77 % more likely to acquire HIV than abstainers and that heavy episodic drinking doubled infection risk (22, 23). Alcohol impairs judgement and decreases condom negotiation, leading to more unprotected sex (22). A multicountry survey among men who have sex with men (MSM) in India reported that frequent alcohol use was associated with being aged ≥25 years and ever married, and was linked to inconsistent condom use with male partners (24). These findings mirror our cohort's characteristics, suggesting that alcohol consumption may be both a social norm and a driver of risk in Kazakhstan.

Nearly two-thirds (67.5 %) of participants reported lifetime use of illicit drugs, and 97 % of these had injected. IDU remains a major transmission route globally; more than half of IDUs in some settings are diagnosed late (25, 26). IDU often co-occurs with social marginalization, poverty and stigma, which delay healthcare seeking (25). In our study, however, IDU was associated with higher odds of early infection (discussed below), indicating that local harm-reduction initiatives or symptom-driven testing may have reached this population.

HIV self-testing was used by 80.8 % of participants before confirmatory diagnosis. This is remarkable given that self-testing is a relatively new strategy in many low- and middle-income countries. Studies in Cambodia and other Asian settings have shown that self-testing is highly acceptable among key populations: in Cambodia, more than 70 % of MSM, transgender women and entertainment workers who self-tested had never previously tested and nearly all reacted to positive results by seeking confirmatory testing and care (27). In San Diego–Tijuana, 81 % of people who inject drugs were willing to use and distribute self-test kits, and willingness was higher among those with larger social networks (28). The high uptake in our cohort may reflect national policies promoting self-testing or the implementation of community-based distribution channels.

STIs were reported by only 5.8 % of participants, with a sharp decline from 9.4 % in 2023 to 1.0 % in 2024. STIs increase HIV susceptibility by disrupting mucosal barriers and inducing inflammation (10, 29). The low prevalence may indicate successful STI management or under-reporting due to stigma and limited diagnostic capacity. Because STIs amplify HIV transmission, integrated services remain essential to sustain gains (10).

### 4.3 Temporal changes from 2023 to 2024

Between 2023 and 2024, the proportion of early HIV infections declined from 27.4 % to 16.2 %. Simultaneously, self-reported alcohol and drug use decreased substantially, and STI diagnoses plummeted. These trends may signal behavioral changes driven by public health campaigns or economic disruptions. Alternatively, they could reflect changes in testing patterns; if high-risk individuals were tested and diagnosed earlier in 2023, those diagnosed later may have been people with slower disease progression. National data from Iran and China have shown modest declines in late diagnosis over time, attributed to expanded testing and ART access (21, 30). However, late diagnosis remains common; in Iran, more than half of newly diagnosed patients are late presenters, particularly older men and IDUs (21). In contrast, the decrease in early infection in our cohort might suggest that fewer new infections occurred or that more infections progressed beyond 6 months before diagnosis. The slight increase in the mean age from 40.8 to 42.9 years and the stability of viral load and CD4 counts support the latter interpretation.

Alcohol use declined from 84.3 % to 66.7 %, and lifetime drug use from 38.2 % to 25.3 % between the 2 years, yet the proportion of injectors among drug users increased. An Ethiopian case–control study found that frequent alcohol users were 3.6 times more likely to present late to care (31), highlighting the importance of addressing alcohol use to facilitate timely testing. The decline in alcohol and drug use in our cohort could reflect social desirability bias in self-reports, mortality among heavy users or improved harm-reduction services. Meanwhile, the rising proportion of injection may signify a shift toward more harmful drug use patterns, underscoring the need for targeted interventions.

STI diagnoses decreased sharply, coinciding with global scaling-up of STI treatment and partner notification services. Community-based testing and treatment may have reduced STI prevalence and, consequently, the contribution of ulcerative infections to HIV transmission. Nonetheless, the near absence of STIs in 2024 is unlikely given the epidemiology of HIV and may reflect under-diagnosis.

### 4.4 Determinants of early vs. long-term infection

Age was modestly associated with early infection in the unadjusted models but lost significance after adjusting for behavioral factors. Older age has been consistently associated with late diagnosis and lower baseline CD4 counts (30, 32). In Iran, individuals aged ≥40 years were 4–8 times more likely to be late presenters than those aged 15–29 years (21). Our study found that older age predicted higher viral loads and lower CD4 counts but did not independently predict infection stage, suggesting that behavioral factors mediate the effect of age on diagnosis timing.

Male gender was associated with greater odds of early infection (OR = 0.58 for long-term infection). This contrasts with large cohorts in Iran and China where men were more likely to be diagnosed late (21, 30). Several factors may explain this divergence. First, Kazakhstan has implemented male-focused outreach through harm-reduction services and community testing, which may have improved timely diagnosis among men. Second, men in our cohort may engage in behaviors (e.g., injection drug use) that prompt earlier testing due to symptom onset or interaction with healthcare services. Third, the small proportion of men (35.4%) in the sample reduces precision, and the association may not generalize.

In multivariable logistic regression, alcohol use (OR = 0.37) and ever drug use (OR = 0.48) were inversely associated with long-term infection, indicating that users were more likely to be diagnosed within 6 months of infection. This finding is counterintuitive given extensive evidence that alcohol and drug use delay testing and reduce ART adherence (11). The narrative review by Williams et al. notes that alcohol use impedes HIV testing and engagement in care and is associated with faster disease progression (11). In Ethiopia, frequent alcohol users presented late (31). One possible explanation for our finding is that people with heavy alcohol or drug use are more likely to experience symptoms of seroconversion or opportunistic infections that trigger early testing. Alternatively, harm-reduction and outreach programs may have targeted drinkers and drug users for recency testing, thus improving early detection. Our measure of alcohol use was binary (“any” vs. “none”) and may not capture the severity or chronicity of use. The small negative association between alcohol servings per day and long-term infection (*B* = −0.001) suggests that heavier drinkers may be diagnosed even earlier, perhaps due to frequent healthcare encounters for alcohol-related conditions.

Having more sexual partners increased the odds of long-term infection; each additional partner was associated with a 75 % rise in the odds of being diagnosed after 6 months. This finding contrasts with studies among MSM in San Diego, where early/acute infection was associated with more male partners and condomless receptive anal intercourse (33), and with general evidence that multiple concurrent partnerships accelerate transmission (10). Our cohort comprises predominantly heterosexual men and women; those with many partners may be part of hidden networks with poor healthcare access and experience chronic infections that go undiagnosed for longer. They may also face higher levels of stigma, discouraging early testing. Moreover, concurrency and partner turnover increase the probability of repeated exposures but may not necessarily prompt individuals to test unless symptoms arise. The borderline trend suggesting higher condom use among early infection cases aligns with the possibility that those who test early are also more health-aware.

Any STI within the past 6 months was more common among early infection cases. Biological synergy between STIs and HIV is well-documented; ulcerative diseases facilitate HIV entry, and non-ulcerative infections increase viral shedding and infectiousness (10, 34). Individuals with STIs may be more likely to seek testing because of symptoms, resulting in earlier HIV diagnosis. Alternatively, recency testing algorithms may classify recent infections when seroconversion coincides with STI diagnosis. Our findings underscore the importance of integrating STI and HIV services.

HIV self-testing was significantly more frequent among early infection cases, consistent with evidence that self-testing improves early detection. In a study comparing self-testing with facility-based testing among MSM, the proportion of early infection was almost twice as high in the self-testing group (35). The Polish overview of self-testing notes that early detection and initiation of ART improve prognosis and reduce transmission (36). Our results suggest that self-testing not only increases overall diagnosis but also shifts detection toward the early stage. This supports recommendations by the World Health Organization and others to expand self-testing for populations that may avoid facility-based services (27).

While lifetime drug use predicted early infection, the proportion of injection among drug users increased over time. IDU is linked to complex networks of risk; it is associated with trading sex, methamphetamine use and exposure to violence (37). Recent surveys among people who inject drugs in Cambodia found that being female, older and homeless increased the odds of HIV infection (38). In our cohort, nearly all drug users injected, and more than half shared needles. The apparent paradox of IDUs being more likely to present early could be explained by targeted harm-reduction programs (e.g., needle exchange) that provide testing or by the onset of opportunistic infections prompting medical attention. The high willingness of PWID to use and distribute self-testing kits may also contribute to earlier diagnoses (28). Nevertheless, the rising proportion of injection among drug users calls for intensified harm-reduction and substitution therapy to prevent both HIV transmission and other blood-borne infections.

### 4.5 Predictors of viral load and CD4 count

Older age was independently associated with higher viral load and lower CD4 count at diagnosis. This is consistent with previous studies demonstrating that older individuals have more advanced disease at presentation and slower immunological recovery (32, 39). In southern Iran, each year of age was associated with a decrease of 112 CD4 cells/μl and an increase in the odds of advanced HIV disease (32). Aging attenuates immune regenerative capacity and is often accompanied by comorbidities and delays in testing, explaining the observed associations. Our finding reinforces the need for age-specific testing strategies and early ART initiation in older adults.

Early infection was associated with higher CD4 counts but not with lower viral loads. Recent infection has been linked to better immunological status because CD4 depletion is minimal during the initial months of infection. A longitudinal analysis of the MERLIN study showed that immediate ART initiation during primary infection significantly reduced the viral reservoir and preserved immune function (35). However, viral load peaks during acute infection and may not differ markedly from chronic infection until several months after seroconversion. The absence of an association between behavioral factors and baseline viral load or CD4 count suggests that demographic variables and infection stage play a more prominent role than substance use in determining immunological status at diagnosis.

### 4.6 Limitations of this study

This study has several limitations. First, its retrospective design and reliance on self-reported behaviors introduce recall and social desirability bias. Alcohol and drug use may be under-reported, and the timing of exposure relative to HIV infection is uncertain. Second, we classified infection stage using recency testing algorithms and self-reported exposure history; misclassification could occur if serological assays misidentify long-term infections as recent or vice versa. Third, our sample was drawn from a single regional center, limiting generalizability to other settings within Kazakhstan and Central Asia. Fourth, approximately 12 % of viral load and CD4 measurements were missing, which may bias regression estimates if missingness is related to unobserved factors. Fifth, we could not account for mental health disorders or socio-economic variables such as income and housing, all of which may influence HIV outcomes. Finally, the cross-sectional nature of the baseline data precludes causal inference; associations should be interpreted cautiously and require confirmation in prospective studies. Moreover, the number of early cases (n=54) limits detection of modest associations. *Post-hoc* detectability analysis indicates that only large effects (e.g., OR ≳ 2.5 when control prevalence ≈ 0.35) are reliably detectable; therefore, non-significant results and wide CIs are expected, and subgroup analyses were purposefully restricted.

Missingness in VL and CD4 was ~0.5% each, and the direction and magnitude of associations between infection stage and laboratory thresholds remained unchanged under extreme-bounds sensitivity analyses. Given this negligible missingness, complete-case inference is appropriate here, and any residual bias from departures from MAR would be inconsequential for our conclusions.

### 4.7 Future directions

Given the high burden of alcohol and drug use in this cohort, integrating harm-reduction, addiction treatment and mental health services into HIV care is imperative. Interventions should address the social determinants that drive substance use and risky sexual behaviors, such as poverty, gender inequality and stigma. Prospective longitudinal studies across multiple regions in Kazakhstan and neighboring countries are needed to validate our findings and explore causal pathways. Such cohorts should collect detailed information on the frequency and patterns of alcohol and drug use, incorporate biomarkers of recent infection and substance use, and evaluate the impact of interventions.

Scaling up HIV self-testing and recency testing among key populations could accelerate early diagnosis. Evidence from Cambodian and PWID studies suggests that self-testing is both acceptable and feasible (27, 28). Policies that subsidize or distribute self-test kits through pharmacies, harm-reduction programs and community organizations may enhance reach. At the same time, linkage to confirmatory testing and care must be strengthened to ensure that early detection translates into improved outcomes. Recency testing should be integrated into national surveillance systems to better estimate incidence and guide prevention resources.

Finally, future research should examine the role of sexual network dynamics and partner concurrency in shaping the timing of HIV diagnosis. Mathematical modeling and social network analyses could identify clusters of late diagnosis and inform targeted outreach. Qualitative studies may elucidate why individuals with multiple partners tend to be diagnosed later, exploring stigma, risk perception and healthcare engagement. By combining quantitative and qualitative methods, researchers can develop culturally sensitive interventions that address both behavioral and structural determinants.

## 5 Conclusion

The cohort findings demonstrate that targeted outreach can markedly influence the stage at which HIV is diagnosed. In our study, men, drinkers and individuals with a history of drug use were more likely to be diagnosed within 6 months of infection, implying that harm-reduction and recency-testing initiatives are succeeding at reaching substance-use networks. By contrast, older adults and those reporting more sexual partners tended to present late and exhibited higher viral loads and lower CD4 counts, highlighting a persistent gap in engagement for these groups. Collectively, the results call for integrated, age-sensitive prevention strategies that unite self-testing, sexual health services and substance-use care, and for intensified community-based education aimed at adults who may underestimate their risk. Expanding self-testing distribution, enhancing linkage to care and continuing to scale up harm-reduction programs will be vital for reducing HIV morbidity and transmission in Kazakhstan and comparable settings worldwide.

## Data Availability

The raw data supporting the conclusions of this article will be made available by the authors, without undue reservation.
