# Supplementary material for: Socio-behavioral determinants of early vs. long-term HIV infection in Kazakhstan: roles of self-testing, alcohol, drug use and sexual networks
Source: Front Public Health. 2025 Oct 28;13:1697913. doi: 10.3389/fpubh.2025.1697913 (PMC12602403; doi:10.3389/fpubh.2025.1697913)
Supplement: Supplementary file 1 [file Supplementary_file_1.docx]

Supplementary Material

# Supplementary Table 1. Early vs. Long-term HIV; counts, ORs, and 80% power thresholds

| Exposure | Early Yes/No | Long-term Yes/No | Control prev (p₂) | Case prev (p₁ obs) | Crude OR [95% CI] | Adjusted OR [95% CI] | Detectable OR @80% power (two-sided), given p₂ |
| --- | --- | --- | --- | --- | --- | --- | --- |
| Alcohol last year | 44/10 | 117/26 | 81.8% | 81.5% | 0.98 [0.44, 2.19] | 1.02 [0.44, 2.34] | ≥ 4.58 (p₁ = 0.95); ≤ 0.36 (p₁ = 0.62) |
| Ever drug use | 26/28 | 50/93 | 35.0% | 48.1% | 1.73 [0.92, 3.26] | 1.46 [0.75, 2.81] | ≥ 2.47 (p₁ = 0.57); ≤ 0.35 (p₁ = 0.16) |

# Supplementary Table 2. Missingness for VL and CD4 (overall and by stage), 2×2 counts for HIGH-VL ≥ 100,000 and LOW-CD4 < 200, crude ORs with 95% CIs and p-values, and extreme-bounds sensitivity estimates (worst-case and best-case).

| Exposure | a | b | c | d | OR (complete-case) | 95% CI | Worst-case OR† | 95% CI | Best-case OR‡ | 95% CI |
| --- | --- | --- | --- | --- | --- | --- | --- | --- | --- | --- |
| HIGH-VL (≥100,000) | 19 | 35 | 74 | 68 | 0.499 | 0.261–0.954 | 0.506 | 0.265–0.967 | 0.492 | 0.258–0.941 |
| LOW-CD4 (<200) | 1 | 52 | 31 | 112 | 0.069 | 0.009–0.523 | 0.139 | 0.032–0.603 | 0.068 | 0.009–0.513 |

Footnote:

HIGH_VL = VL ≥ 100,000 copies/mL; LOW_CD4 = CD4 < 200 cells/µL.

2×2 counts are: a = EARLY=1 & exposure=1, b = EARLY=1 & exposure=0, c = EARLY=0 & exposure=1, d = EARLY=0 & exposure=0.

† Worst-case: set missing values to 1 for EARLY=1 and 0 for EARLY=0.

‡ Best-case: set missing values to 0 for EARLY=1 and 1 for EARLY=0.
